# Measuring meaning-based well-being in individuals with dementia: the creation and validation of the well-being in dementia inventory

**DOI:** 10.1093/ageing/afaf092

**Published:** 2025-04-16

**Authors:** Zehra B Turel, Allan Perry, Alexander Balicki, Elizabeta Mukaetova-Ladinska, Estefania Vargas Triguero, Anna Lesniak, John Maltby

**Affiliations:** PJ Care, Eagle Wood Neurological Care Centre, Bretton Way, Peterborough, PE3 8AQ, UK; PJ Care, Eagle Wood Neurological Care Centre, Bretton Way, Peterborough, PE3 8AQ, UK; PJ Care, Eagle Wood Neurological Care Centre, Bretton Way, Peterborough, PE3 8AQ, UK; University of Leicester, College of Life Sciences, University Road, Leicester, Leicestershire, LE1 7RH, UK; PJ Care, Eagle Wood Neurological Care Centre, Bretton Way, Peterborough, PE3 8AQ, UK; PJ Care, Eagle Wood Neurological Care Centre, Bretton Way, Peterborough, PE3 8AQ, UK; University of Leicester, College of Life Sciences, University Road, Leicester, Leicestershire, LE1 7RH, UK

**Keywords:** dementia, eudaimonic, meaning based, scale validation, holistic care, older people

## Abstract

Despite growing attention to well-being in dementia, few studies have defined meaning-based (eudaimonic) well-being in this population, mainly due to challenges posed by cognitive decline and self-report limitations. We developed and validated a novel tool for measuring meaning-based well-being in individuals with dementia, particularly those receiving residential or home care. The study included two samples: carers of 174 care home residents and carers of 420 community-dwelling individuals for whom respondents reported dementia. The Well-being in Dementia Inventory (WiDI) assesses six core dimensions: Self-Sufficiency, Functional Mastery, Goal-Based Mastery, Purposeful Engagement, Positive Interactions and Constructive Self-Perspective. Confirmatory Factor Analysis established the WiDI’s six-factor structure, underscoring its multidimensional nature and equivalence across community-dwelling individuals, regardless of gender, age group (younger-old/mid-older-old), or care context (family or professional). The scale exhibited high internal and inter-rater reliability, though very low scores in the care home sample inflated these statistics. Concurrent validity was confirmed through strong correlations with adapted indices of meaning-based well-being (e.g. the Scales of Psychological Well-being and the Mental Health Continuum Short Form, commonly used in non-dementia samples), indicating the WiDI’s conceptual consistency. These findings clarify how meaning-based well-being can be assessed in individuals with dementia and introduce the WiDI as a reliable and valid tool for assessing well-being, suggesting broad applicability across care settings. These results have important implications for practice and policy, advocating a meaning-based approach to well-being assessments that ensures holistic, personalised care by focusing on key indicators of life quality.

## Key Points

- Introduces Well-being in Dementia Inventory (WiDI) to assess eudaimonic well-being in dementia.
- The WiDI assess self-sufficiency, mastery, engagement, interactions and self-perspective.
- The WiDI exhibits structural validity, inter-rater reliability and concurrent validity.
- WiDI effectively addresses dementia well-being challenges, focusing on caregiver insights.
- WiDI promotes well-being in dementia, influencing care strategies and decisions.

## Introduction

The UK Chief Medical Officer urges a shift in dementia care, emphasising quality of life over longevity and advocating ‘less medicine, not more’ [1]. A distinction exists in well-being between affect/symptom-based (hedonic) and meaning-based (eudaimonic) dimensions [2, 3]. Affect/symptom-based well-being is often prioritised by focusing on symptoms such as anxiety and depression, measured by Anxiety and Depression Scales [4] and Quality of Life measures [5]. Well-being in dementia goes beyond symptom management to prioritise dignity, social connections and personalised care. Incorporating meaning-based well-being into support frameworks requires revised assessment protocols to support meaning-based well-being pathways throughout dementia [6].

Meaning-based well-being is popularly defined by autonomy, environmental mastery, personal growth, purpose in life, positive relationships and self-acceptance [2, 3]. Measures of meaning-based well-being for older adults, including those with dementia, often derive from Ryff’s Psychological Well-being Scales [2], regarded as the gold standard for assessing Autonomy, Environmental Mastery, Personal Growth, Purpose in Life, Positive Relations with Others and Self-Acceptance. These scales are central to understanding well-being across the lifespan and are frequently cited [7, 8]. Other tools, including the Short Warwick-Edinburgh Mental Well-Being Scale [9], integrating symptom-based and meaning-based elements and the Flourishing Scale [10], which highlights social connections and meaningful experiences, have also been applied in older populations. However, Ryff's framework [2, 3] remains comprehensive, with strong validation and application in older age groups. Evidence suggests it fosters resilience against cognitive decline, supports navigating care transitions, strengthens social ties, promotes personal growth, aids in coping with loss and addresses existential struggles in later life [11].

For individuals with dementia, adapting these scales is increasingly recognised as necessary [8]. Communication challenges and cognitive decline make self-report measures impractical, requiring innovative approaches with behavioural indicators and caregiver insights. Adapting Ryff’s scales for third-party assessments [8] fails to capture the complexities of meaning-based well-being in this population. Factors like physical health, mobility and evolving social relationships alter expressions of constructs such as autonomy and environmental mastery. Whilst individuals with dementia may struggle to articulate their experiences, it does not diminish the importance of addressing these dimensions. A new framework for assessing meaning-based well-being, tailored to the challenges faced by those living with dementia, and that utilises the experiences of those caring for the individual is crucial for accurately capturing and supporting well-being in dementia.

This study operationalises the assessment of meaning-based well-being for individuals with dementia, using Ryff’s model as a starting template and using the insights and experiences of those caring for those individuals with dementia. Two objectives guide this effort:

(1) Develop assessment to measure meaning-based well-being in dementia, ensuring reliability and structural validity as a multi-dimensional tool (Objective 1).
(2) Demonstrate the assessment’s construct validity by confirming concurrent validity with established meaning-based well-being measures (Objective 2).

## Method

### Samples

Three samples were studied, comprising carers assessing the individual in their care.

Sample 1 examined the reliability and structural validity (Objective 1) of the introduced measure, using three assessment points from carers of 174 residents (98 males, 76 females; Mean age = 63.84, SD = 13.74) in a UK-based specialist care home focused on individuals with complex neurological conditions and neurodegenerative diseases. Of them, 65.5% were aged over 60. Reported conditions included Alzheimer’s (*n* = 36), unspecified dementia (*n* = 50), brain/spinal cord injuries/disorders (*n* = 45), Huntington’s (*n* = 16), stroke-related (*n* = 8), multiple sclerosis (*n* = 6) and Parkinson’s (*n* = 5), plus seven residents with other conditions. Assessments were not completed for every resident at each time point: Day 1 (*n* = 156), Day 2 (*n* = 145) and Day 3 (*n* = 148).

Sample 2, a subset of Sample 1, focused on inter-rater reliability (Objective 1). Data were collected 3 months later, involving 102 residents.

Sample 3 examined structural (Objective 1) and concurrent validity (Objective 2) with 420 carers (151 males, 269 females; Mean age = 39.80, SD = 12.22) recruited via the crowdsourcing site Prolific. These carers looked after individuals over 60 with dementia, based on their own reports rather than clinical or research confirmation (176 males, 244 females; mean age = 72.15, SD = 8.84). Ethnicity was reported as White (81.4%), Asian (9.3%), Black (5.2%), Mixed Race (2.9%) and Other (1.2%). All respondents confirmed the individual had dementia, with primary diagnoses of Dementia/Alzheimer’s (*n* = 323), chronic illnesses (*n* = 51), or other neurological disorders (*n* = 46). Carers provided an average of 41.44 h (SD = 46.94) of care weekly, with 250 being spouses, family or friends and 170 in a professional role.

### Measures

#### Well-being in dementia inventory

The rationale for the Well-being in Dementia Inventory (WiDI) lies in its departure from the traditional abductive approach, which typically generates item pools aimed at assessing desired constructs, with scales finalised after population testing [12, 13]. In cases where individuals have limited capacity, such scales may include items that do not apply, undermining their relevance for those most in need. Moreover, abductive development often leads to longer assessments, requiring multiple items to cover each construct, which can be impractical when time is restricted. Finally, where the person being assessed cannot communicate, the assessor’s expertise becomes critical. By contrast, the WiDI adopts a more deductive approach and uses composite item construction, focusing on the judgement of care professionals or carers. Rather than assessing each behaviour in isolation, it draws on behavioural indicators and experiences around the care of the individual with dementia. Targeting higher-level constructs of meaning-based well-being (autonomy, environmental mastery, personal growth, purpose in life, positive relationships and self-acceptance), the WiDI aims to capture these dimensions in individuals with dementia through shorter, more focused items. For instance, rather than many questions about personal care tasks, the WiDI includes a single item on personal care (e.g. ability to bath, grooming, dressing), allowing for nuanced evaluation of autonomy without relying on exhaustive lists. By enabling assessors to draw on their experience and judgement, the WiDI promotes broader engagement with well-being constructs and focusses the assessment on core domains of primary importance.

The WiDI was developed over 18 months through an iterative process involving a range of professionals: neuropsychologists, psychiatrists, psychologists, care managers, nurses and care staff. Over 100 potential items were created from clinical experience and existing measures, then piloted with care staff. Factor analysis of pilot data identified overlapping items and produced broader candidate constructs subsuming single items, creating a prototype scale. The potential items were subsequently reviewed with clinical and professional care teams to ensure alignment with practical care needs. Additional iterative feedback rounds and piloting followed, integrating insights from professional teams and care staff to confirm precision, relevance and language use. The final version of the WiDI (See Appendix 1 in the Supplementary Data) assesses six dimensions:

##  

(1) Self-sufficiency (three items), reflecting autonomy in personal care, routine and activities

Two aspects of environmental mastery:

(2) Functional Mastery (three items), addressing physical aspects of functioning; flexibility, strength and coordination.
(3) Goal-Based Mastery (three items), capturing functional and cognitive elements; task performance, focus and adaptability.
(4) Purposeful Engagement (four items) combines Personal Growth and Purpose in Life, emphasising personal goals, diverse activities, time management and group participation.
(5) Positive Interactions (four items) reflects Positive Relations through relationship types, friendliness, compassion and communication quality.
(6) Constructive Self-Perspective (four items) embodies Self-Acceptance via understanding and managing challenges, maintaining a positive self-view, self-belief and self-kindness.

Items are rated from 0 (‘Never’) to 5 (‘Always’). In Sample 1, assessments covered the preceding 24 hours. In Sample 2, two carers or nurses also assessed items over the preceding 24 hours. Sample 3 used a month-long timeframe for evaluation, accommodating those providing intermittent care.

### Meaning-based well-being measures

Sample 3 participants were assessed using modified versions of the Scales of Psychological Well-being [2] and the Psychological Well-being Subscale of the Mental Health Continuum Short Form [14]. Both scales typically measure eudaimonic well-being in the general population, focusing on six dimensions. Following van Herwaarden *et al.* [8], who adapted these measures for individuals with dementia, items were rephrased in the third person. Whilst neither scale is fully validated for dementia nor ideal for establishing concurrent validity, they remain recognised references for assessing meaning-based/eudaimonic well-being. Although not directly comparable, they offer a valuable benchmark for assessing the WiDI. By operationalising meaning-based well-being through items reflecting broader meaning-based/eudaimonic descriptions, this approach demonstrates concordance and conceptual consistency, supporting the WiDI’s concurrent validity.

### Data analysis

We used Cronbach's alpha coefficients to assess internal reliability (Objective 1), which demonstrated satisfactory results. Alpha criteria were: α < 0.5 = Unacceptable; 0.5 ≤ α < 0.6 = Poor; α ≥ 0.6 = Acceptable; α ≥ 0.7 = Good [15]. Inter-rater reliability was examined via intra-class correlations (ICC) between pairs of carers (Sample 2) using a One-Way Random model, chosen due to varying raters. ICC ≥ 0.6 indicated ‘good,’ and ICC ≥ 0.75 ‘excellent’ [16].

We conducted Confirmatory Factor Analysis (CFA) to examine the structural validity (Objective 1) of the WiDI. CFA tests whether data fit a hypothesised measurement model. For Sample 1, we used Unweighted Least Squares (ULS), appropriate for non-normally distributed data (skewness> ± 2) [17]. For Sample 3, we applied Maximum Likelihood (ML) for normally distributed data. Because ULS and ML require different goodness-of-fit measures [18–21], we checked for ULS, Goodness-of-Fit Index (GFI) and Normed Fit Index Fit Index (NFI) ≥ 0.95, Adjusted Goodness of Fit (AGFI) ≥ 0.90, Standardised Root Mean Squared Residual (SRMR) < 0.08; and for ML, Comparative Fit Index (CFI) and Non-Normed Fit Index (NNFI) ≥ 0.90, and Root Mean Square Error of Approximation and SRMR<0.08. As recommended when demonstrating the value of models [22], we compared the incremental value of a six-factor interpretation of the data against a one-factor model in which all items load on a single latent factor of meaning-based well-being. For the ML estimation, a difference in CFI ≥ 0.01 indicates an improved fit [27]. However, for ULS, no similar criteria have been proposed, so we rely on comparing improvements in fit indices.

Multi-group CFA (Objective 1) was conducted for subgroups with n > 150 [23, 24], to examine whether the scale functioned consistently regardless of the person’s gender (male/female), age (young-old, 60 or 65–74, and middle/old-old, 75+) [25, 26], or caregiver relationship (family/friend, professional). Increasingly strict constraints (configural, metric, scalar and strict) established invariance if the decrease in CFI was ≤0.010, and increases in RMSEA and SRMR were ≤ 0.015 or ≤ 0.030 (metric), or ≤ 0.010 (scalar/strict) [27].

To establish concurrent validity (Objective 2), Pearson product–moment correlation coefficients were calculated between the WiDI scales and subscales of the Psychological Well-being and Mental Health Continuum Short Form. Effect sizes were interpreted as small (*r* = 0.10), medium (*r* = 0.24) and large (*r* = 0.37) [28].

## Results

Table 1 shows the percentage frequency of items across Sample 1 (Time 1 to 3) and Sample 3. Amongst Sample 1, a significant proportion of responses fall into the ‘0’ category, often exceeding 50% and in some cases reaching over 70%. This pattern indicates a population requiring specialist neurological care. Due to the high prevalence of ‘0’s in Sample 1 (inflating reliability assessments), we computed for this sample reliability and factor analysis statistics for the total sample and a subsample (‘non-omitted’) that excluded individuals who scored 0 across the entire assessment, with those retained exhibiting a score above 0 on at least 1 of the scales. These subsamples represented between 72.4% and 77.2% of the original samples from Time 1 to Time 3.

**Table 1:** Frequency table of percentage of response category to each item by each sample.

	Sample 1 (Time 1) (*n* = 156)^a^						Sample 1 (Time 2) (*n* = 145)^a^					
	%						%					
	Never					Always	Never					Always
Item	0	1	2	3	4	5	0	1	2	3	4	5
1	68.6	10.3	3.8	3.8	3.8	9.6	64.8	11.0	9.0	4.8	2.1	8.3
2	63.5	9.6	7.7	3.8	2.6	12.8	57.9	12.4	6.2	6.9	3.4	13.1
3	73.1	12.2	3.8	3.8	1.3	5.8	70.3	12.4	6.2	4.1	1.4	5.5
4	51.3	10.3	6.4	6.4	8.3	17.3	49.0	11.7	2.8	9.7	9.7	17.2
5	63.5	8.3	4.5	8.3	4.5	10.9	58.6	7.6	6.2	9.0	6.2	12.4
6	55.1	9.0	7.1	12.2	6.4	10.3	47.6	12.4	10.3	12.4	6.2	11.0
7	67.3	10.3	7.1	4.5	3.2	7.7	70.3	7.6	7.6	4.8	3.4	6.2
8	76.3	12.8	4.5	1.9	1.9	2.6	75.9	10.3	7.6	2.1	1.4	2.8
9	71.2	10.9	7.7	3.8	0.6	5.8	69.0	9.7	7.6	5.5	2.8	5.5
10	78.2	12.2	3.8	1.9	1.9	1.9	80.7	10.3	3.4	1.4	1.4	2.8
11	72.4	14.1	5.8	1.9	3.8	1.9	74.5	12.4	55.5	4.8	1.4	1.4
12	78.8	10.9	3.2	1.9	1.3	3.8	80.0	9.0	4.8	0.7	1.4	4.1
13	72.4	12.8	7.1	3.2	1.3	3.2	75.2	10.3	6.2	4.8	0.7	2.8
14	54.5	14.7	11.5	9.6	3.2	6.4	48.3	20.0	11.0	9.7	5.5	5.5
15	53.2	16.7	13.5	5.8	3.8	7.1	50.3	15.2	14.5	9.7	3.4	6.9
16	60.9	12.8	10.3	6.4	5.8	3.8	57.2	13.1	9.7	9.7	6.9	3.4
17	58.3	15.4	6.4	9.0	3.8	7.1	58.6	14.5	4.8	9.0	6.9	6.2
18	75.0	10.3	8.3	1.9	1.3	3.2	76.6	6.9	9.7	3.4	1.4	2.1
19	72.4	13.5	5.8	1.9	3.2	3.2	71.0	9.0	12.4	3.4	2.1	2.1
20	72.4	12.8	5.8	3.2	3.2	2.6	75.9	9.7	4.8	5.5	2.1	2.1
21	69.2	13.5	5.8	5.1	3.2	3.2	67.6	9.0	12.4	6.2	2.8	2.1
	Sample 1 (Time 3) (*n* = 148)^a^						Sample 3 (*n* = 420)					
	Never					Always	Never					Always
	0	1	2	3	4	5	0	1	2	3	4	5
1	63.5	12.8	5.4	4.1	2.7	11.5	10.7	12.6	15.0	16.4	24.3	21.0
2	58.8	11.5	8.8	7.4	4.1	9.5	7.9	13.3	16.7	19.8	23.8	18.6
3	68.9	9.5	8.8	5.4	2.0	5.4	9.3	12.1	19.5	21.7	23.8	13.6
4	54.1	8.1	6.8	5.4	6.8	18.9	6.4	15.2	19.8	21.9	16.7	20.0
5	61.5	8.8	4.1	8.1	4.7	12.8	8.1	20.5	20.5	22.4	15.5	13.1
6	52.7	10.8	7.4	12.2	5.4	11.5	6.2	16.7	22.4	23.6	17.4	13.8
7	65.5	11.5	6.1	6.1	2.0	8.8	7.6	8.8	15.5	19.5	24.5	24.0
8	76.4	8.8	7.4	4.1	0.7	2.7	8.3	11.9	22.9	25.7	18.6	12.6
9	68.2	13.5	5.4	6.1	2.0	4.7	6.0	11.2	17.6	20.5	25.5	19.3
10	85.1	8.1	3.4	0.7	1.4	1.4	14.5	21.9	17.9	23.6	14.8	7.4
11	75.7	12.8	6.1	3.4	0.7	1.4	15.0	25.0	22.4	21.4	11.4	3.8
12	80.4	10.1	3.4	1.4	0.7	4.1	12.1	20.5	20.7	23.6	15.0	8.1
13	73.6	11.5	7.4	2.7	2.0	2.7	19.8	26.7	18.3	19.5	11.4	4.3
14	48.6	15.5	9.5	14.2	6.8	5.4	1.2	7.9	11.0	21.4	31.7	26.9
15	52.7	10.8	10.8	12.2	4.7	8.8	0.5	6.4	12.6	21.4	32.6	26.4
16	56.8	12.2	10.1	8.8	8.1	4.1	1.9	6.4	13.8	21.9	30.0	25.5
17	56.8	9.5	7.4	10.1	8.1	8.1	0.7	11.7	13.8	24.0	30.5	19.3
18	75.7	6.1	9.5	4.1	3.4	1.4	3.3	14.0	21.2	27.6	23.3	10.5
19	77.7	6.1	6.8	6.1	1.4	2.0	6.2	14.3	20.5	30.0	19.3	9.8
20	73.6	10.8	4.7	6.8	2.7	1.4	4.3	16.7	21.2	27.9	18.6	11.4
21	67.6	8.8	8.1	8.8	4.7	2.0	6.7	15.7	20.5	24.8	21.2	11.2

^a^These data are drawn from a larger set of the same 174 residents, with each sample number indicating the number of completed assessments conducted on a given day.

In terms of reliability (Objective 1), Table 2 outlines the Cronbach’s alpha coefficients for internal reliability, none considered unacceptable. Generally, these coefficients meet the ‘good’ threshold. However, the Sufficiency subscale in Sample 1 at the second assessment would be considered ‘acceptable’ for the total sample, and poor to acceptable for the second and third administrations in the ‘non-omitted’ sample. For inter-rater reliability in Sample 2 (covering both the total and ‘non-omitted’ samples), results showed satisfactory reliability, with ICC reaching or exceeding 0.82, indicating high agreement amongst raters. Mean scores for all subscales are presented for Samples 1 and 3.

**Table 2:** Cronbach’s alpha, minimum to maximum scores, mean (divided by number of items), standard deviation, skewness and kurtosis statistics for the WiDI scale for the resident (total and non-omitted sample) and community samples.

				Sample 1 (Time 1) (*n* = 156)^a^			
	α^b^	α^c^	Min–Max	Mean	SD	Skewness	Kurtosis
Self-Sufficiency	.76	.71	0–5	0.90	1.32	1.51	1.31
Functional Mastery	.92	.90	0–5	1.38	1.73	0.99	−0.53
Goal-Based Mastery	.88	.87	0–5	0.69	1.22	2.11	3.92
Purposeful Engagement	.88	.86	0–5	0.51	0.97	2.46	6.37
Positive Interactions	.94	.92	0–5	1.06	1.40	1.34	0.87
Constructive Self-Perspective	.97	.97	0–5	0.61	1.17	2.22	4.31
				Sample 1 (Time 2) (*n* = 145)^a^			
	α			Mean	SD	Skewness	Kurtosis
Self-Sufficiency	.63	.55	0–5	0.96	1.20	1.16	0.57
Functional Mastery	.91	.89	0–5	1.52	1.74	0.82	−0.82
Goal-Based Mastery	.83	.81	0–5	0.71	1.18	1.74	2.23
Purposeful Engagement	.83	.82	0–5	0.48	0.90	2.64	8.38
Positive Interactions	94	.93	0–5	1.14	1.43	1.17	0.24
Constructive Self-Perspective	.94	.93	0–5	0.61	1.08	2.05	3.88
				Sample 1 (Time 3) (*n* = 148)^a^			
	α			Mean	SD	Skewness	Kurtosis
Self-Sufficiency	.73	.65	0–5	0.99	1.30	1.36	1.12
Functional Mastery	.90	.88	0–5	1.42	1.74	0.91	−0.69
Goal-Based Mastery	.81	.78	0–5	0.73	1.17	1.75	2.39
Purposeful Engagement	.81	.80	0–5	0.43	0.83	2.71	8.70
Positive Interactions	.94	.92	0–5	1.26	1.51	0.97	−0.32
Constructive Self-Perspective	.93	.93	0–5	0.62	1.11	1.87	2.62
				Sample 3 (*n* = 420)			
	α			Mean	SD	Skewness	Kurtosis
Self-Sufficiency	.85		0–5	2.89	1.38	−.465	−0.75
Functional Mastery	.91		0–5	2.71	1.37	−.028	−0.88
Goal-Based Mastery	.86		0–5	2.98	1.32	−.401	−0.49
Purposeful Engagement	.90		0–5	2.13	1.27	.162	−0.81
Positive Interactions	.91		0–5	3.48	1.13	−.649	−0.32
Constructive Self-Perspective	.91		0–5	2.75	1.20	−.214	−0.55

^a^These data are drawn from a larger set of the same 174 residents, with each sample number indicating the number of completed assessments conducted on a given day.

^b^Cronbach’s alpha recomputed for resident samples with those scoring 0 across all scales excluded; Time 1, *n* = 113; Time 2, *n* = 112; Time 3, *n* = 109.

^c^Cronbach’s alpha for all persons in each sample.

Regarding structural validity (Objective 1), Table 3 presents the CFA results, indicating a satisfactory overall fit for the six-factor model, aside from a deviation in the second and third (for the non-omitted sample) administration’s SRMR. Moreover, the six-factor model demonstrates improved fit statistics compared to the unidimensional model, which exhibited only acceptable GFI, AGFI and NFI values for the first administration in the resident sample. Table 4 shows the multi-group CFA results, testing measurement invariance across gender (male or female), age group (young-old or middle/old-old) and type of carer (family/friend or professional) for Sample 3. Comparisons were made between male and female individuals being cared for, younger and older individuals and family/friend versus professional carers. The model shows excellent fit with minimal changes in fit indices (ΔCFI, ΔRMSEA, ΔSRMR), signifying strong measurement invariance across configural, metric, scalar and strict indices.

**Table 3:** Fit statistics to assess model fit of the six-factor model across each sample using unweighted least squares (sample 1: Total and non-omitted sample) and maximum likelihood (sample 3).

	*x*2	*df*	GFI	AGFI	NFI	SRMR
	Unweighted Least Squares					
	Total Sample					
	Resident Time 1 (*n* = 156)					
Unidimensional	2073.21		.964	.955	.957	.104
Six-factor	593.37		.988	.986	.988	.064
	Resident Time 2 (*n* = 145)					
Unidimensional	2988.12		.928	.912	.912	.127
Six-factor	1023.02		.975	.967	.970	.086
	Resident Time 3 (*n* = 148)					
Unidimensional	2689		.941	.928	.928	.119
Six-factor	791.11		.983	.977	.979	.076
	Non-Omitted Sample					
	Resident Time 1 (*n* = 113)					
Unidimensional	2603.525		.946	.934	.936	.112
Six-factor	778.901		.984	.979	.981	.070
	Resident Time 2 (*n* = 112)					
Unidimensional	3475.709		.898	.875	.870	.131
Six-factor	1267.987		.963	.950	.953	.092
	Resident Time 3 (*n* = 109)					
Unidimensional	3406.314		.903	.881	.875	.129
Six-factor	1049.898		.970	.960	.961	.084
	Maximum Likelihood					
	Non-Resident (*n* = 420)					
	*x*2	*df*	CFI	NNFI	RMSEA	SRMR
Unidimensional	3686.24	189	.451	.440	.210	.158
Six-factor	494.47	174	.950	.939	.066	.046

Key: *χ*2 = Chi-square; df = degrees of freedom (not given for Unweighted Least Squares); Goodness of Fit Index = GFI; Adjusted Goodness of Fit Index = AGFI; NNFI = Non-Normed Fit Index; CFI = comparative fit index; NNFI = Non-Normed Fit Index/Tucker-Lewis Index; RMSEA = Root Mean Square Error of Approximation; SRMR = Standardised Root Mean Square.

**Table 4:** Multi-group confirmatory factor analysis by broad estimates of gender, age and relationship type of the person cared for (sample 3).

	*χ*2	*df*	CFI	RMSEA	SRMR	ΔCFI	ΔRMSEA	ΔSRMR
	Gender of person (Male, *n* = 176; Female, *n =* 244).							
Configural	684.053	348	0.948	0.068	0.050			
Metric	693.674	363	0.948	0.066	0.052	0.000	−0.002	0.002
Scalar	714.496	378	0.947	0.065	0.053	0.001	−0.001	0.001
Strict	729.322	399	0.948	0.063	0.053	0.001	−0.002	0.000
	Age of the person (60–74 years, *n =* 261; 75+ years, *n =* 159)							
Configural	744.761	348	0.938	0.074	0.053			
Metric	769.669	363	0.936	0.073	0.055	0.002	−0.001	0.002
Scalar	777.056	378	0.937	0.071	0.055	0.001	−0.002	0.000
Strict	816.473	399	0.934	0.071	0.054	0.003	0.000	−0.001
	Relationship to person (Friend/Family, *n* = 250; Professional, *n* = 170)							
Configural	710.781	348	0.944	0.070	0.051			
Metric	722.082	363	0.944	0.069	0.054	0.000	−0.001	0.003
Scalar	731.223	378	0.945	0.067	0.054	0.001	−0.002	0.000
Strict	756.395	399	0.945	0.065	0.054	0.000	−0.002	0.000

Key: *χ*2 = Chi-square; df = degrees of freedom; CFI = comparative fit index; NNFI = Non-Normed Fit Index/ Tucker-Lewis Index; RMSEA = Root Mean Square Error of Approximation; SRMR = Standardised Root Mean Square Residual; Δ = Change.

Regarding concurrent validity (Objective 2), Table 5 shows the correlation matrix between the WiDI and other measures of meaning-based/eudaimonic well-being for Sample 3, revealing strong associations with subscales from the Scales of Psychological Well-being and the Mental Health Continuum Short Form. Correlation coefficients ranged from *r* = 0.515 to *r* = 0.732, suggesting a large effect size between similarly represented well-being domains across scales.

**Table 5:** Pearson product moment correlation coefficients between the WiDI subscales and the scales of psychological well-being (SPW) and mental health continuum short form (MHC) (sample 3).

	Self-Sufficiency	Functional Mastery	Goal-Based Mastery	Purposeful Engagement	Positive Interactions	Constructive Self-Perspective
Autonomy (SPW)	.626^**^	.159^**^	.435^**^	.368^**^	.253^**^	.298^**^
Autonomy (MHC)	.603^**^	.137^**^	.430^**^	.411^**^	.338^**^	.355^**^
Environmental Mastery (SPW)	.457^**^	.653^**^	.664^**^	.510^**^	.294^**^	.423^**^
Environmental Mastery (MHC)	.501^**^	.604^**^	.642^**^	.511^**^	.286^**^	.385^**^
Personal Growth (SPW)	.238^**^	.289^**^	.195^**^	.682^**^	.280^**^	.466^**^
Personal Growth (MHC)	.247^**^	.259^**^	.218^**^	.576^**^	.288^**^	.410^**^
Purpose in Life (SPW)	.240^**^	.196^**^	.205^**^	.603^**^	.217^**^	.257^**^
Purpose in Life (MHC)	.219^**^	.197^**^	.210^**^	.515^**^	.178^**^	.171^**^
Positive Relations with Others (SPW)	.189^**^	−.027	.195^**^	.221^**^	.730^**^	.452^**^
Positive Relations with Others (MHC)	.169^**^	−.015	.171^**^	.239^**^	.732^**^	.480^**^
Self-Acceptance (SPW)	.101^*^	.024	.102^*^	.182^**^	.418^**^	.552^**^
Self-Acceptance (MHC)	.237^**^	.130^**^	.211^**^	.303^**^	.405^**^	.590^**^

Key: ^*^P < 0.05;

^**^ *P* < 0.01; SPW = Scales of Psychological Well-being; MHC = Mental Health Continuum Short Form. The shaded areas indicate the domains of well-being that are similarly represented across scales.

## Discussion

The findings underscore a mostly successful operationalisation of meaning-based well-being for individuals with dementia or respondent-reported dementia through the WiDI using carer reports. This study expands on Ryff’s conceptual foundations by identifying and validating six key dimensions: Self-Sufficiency, Functional Mastery, Goal-Based Mastery, Purposeful Engagement, Positive Interactions and Constructive Self-Perspective. Evidence from the insights and experience of carers of care home residents (Samples 1 and 2) and those living in the community with respondent-reported dementia (Sample 3) indicates that the WiDI can be used and is needed to assess critical aspects of autonomy, cognitive and functional capacities related to environmental mastery, interpersonal dynamics, levels of purpose and self-acceptance amongst populations displaying mild to severe levels of dementia. There is evidence for internal reliability and inter-rater agreement around the use of the WiDI, as well as evidence of measurement invariance of the six-factor model across gender, age groups and carer relationships. The findings support the scale’s consistency and potential adaptability in formal and community dementia care settings where levels of dementia may differ. Furthermore, large-effect-size correlations with established meaning-based measures in Sample 3 demonstrate the WiDI’s concurrent validity and potential to offer a more holistic assessment of well-being, one that encompasses personal growth, dignity and social engagement alongside traditional symptom-focused approaches.

However, some results should be interpreted with appropriate caution. The prevalence of zeros amongst neurological patients with dementia in the first sample underscores the challenges this group faces performing tasks independently. On the one hand, these scores highlight the need for carer assessments and the tool itself, given that most individuals (72–77%) with complex neurological conditions scored above zero on at least one dimension. Yet the high level of zeros in the care sample (regardless of whether we omitted those who scored zero across all scales) inflated internal consistency and inter-rater reliability statistics, reflecting agreement driven by shared tendencies to assign zero scores. Nonetheless, the six-factor structure (and its superior fit over a unidimensional model, particularly in the community sample) alleviates concerns created from inflated reliability, demonstrating six distinct factors. If zeros created a single latent dimension of well-being, a unidimensional solution would be superior. Instead, the six-factor model’s superior fit suggests it captures discrete aspects of well-being, even with prevalent low scores. This finding underscores the instrument’s utility for nuanced assessments requiring carer-based insights. Finally, the WiDI’s subscales show robust correlations with respective established meaning-based/eudaimonic measures, demonstrating conceptual consistency and alignment with distinct aspects of well-being.

The WiDI represents a change in measuring well-being in individuals with dementia by integrating behavioural indicators and carer experience to provide a multidimensional perspective. Using Ryff’s model [2, 3] to encompass meaning-based well-being, it encourages care settings to address not only physical and symptomatic needs but also crucial psychological and social dimensions. The measure considers each dimension to tackle dementia-specific challenges. For instance, Self-Sufficiency (Autonomy) emphasises personal care and daily decision-making, whereas Ryff’s autonomy focuses on self-determination. Functional and Goal-Based Mastery extend Environmental Mastery, reflecting well-being around mobility and task performance. Constructive Self-Perspective reconsiders Self-Acceptance in a care setting to emphasise self-kindness and managing challenges associated with cognitive decline. This novel approach fosters meaning-based well-being, offering a comprehensive tool relevant across the spectrum of cognitive decline, addressing the unique needs of individuals with dementia and encouraging personalised, compassionate care strategies.

Although designed for those with advanced dementia who need proxy ratings, amongst the respondent-reported dementia sample, findings (where responses to items are more varied) confirm the scale’s flexibility for milder impairments. The WiDI’s use in both care-home and community settings suggests it has versatility: it can compare well-being across diverse contexts, assess cultural variations in dementia care and evaluate differences between residential and community environments. Moreover, the WiDI has the potential to serve as an outcome measure in intervention studies, enabling researchers to assess the effectiveness of strategies aimed at improving well-being. By identifying domains such as autonomy and purposeful engagement, the tool supports personalised care planning, resource optimisation, and interventions that foster meaningful activities and relationships. In professional settings, the WiDI offers actionable insights to improve care delivery, whilst in community care, it provides families with a structured way to monitor well-being at home. These capabilities underscore its practical and policy implications, advocating for a holistic approach that integrates meaning-based assessments into dementia care frameworks [1, 6]. Nevertheless, limitations include a lack of specific clinical and care data (e.g. severity indices, sensitivity, socio-economic factors) impacting the precision and applicability of some of the findings. Also, the focus during item development was primarily on formal care settings, potentially overlooking some informal carer perspectives. Consequently, experiences such as using technology or social media to connect were excluded due to limited relevance in those settings. Future studies will be crucial in addressing these gaps, exploring sensitivity to change and application across cultural and clinical contexts, and ensuring continued refinement of the assessment for broader populations.

In conclusion, the WiDI offers a useful tool for assessing meaning-based well-being in individuals with dementia, providing a reliable and valid means assessment tool. Its use supports the development of more compassionate and effective care strategies.

## Supplementary Material

aa-24-2079-File002_afaf092

## Data Availability

The data and scale coding for the WiDI from the community sample (Sample 3) are available on the Open Science Framework: https://osf.io/a8jpe/. The data from Samples 1 and 2 have not been made available due to their clinical and commercial sensitivity.
