# Supplementary material for: Measuring meaning-based well-being in individuals with dementia: the creation and validation of the well-being in dementia inventory
Source: Age Ageing. 2025 Apr 16;54(4):afaf092. doi: 10.1093/ageing/afaf092 (PMC12001781; doi:10.1093/ageing/afaf092)
Supplement: aa-24-2079-File002_afaf092 [file aa-24-2079-file002_afaf092.docx]

Article Title: Measuring Meaning-Based Well-Being in Individuals with Dementia: The Creation and Validation of the Well-being in Dementia Inventory

**Appendix 1**

**Well-Being in Dementia Inventory (WiDI)**

**© 2025 Imnas Health. All rights reserved.**

Items reproduced by Permission of Imnas Health. All rights reserved. The scale items presented in this supplementary material are the exclusive property of Imnas Health. Unauthorised use, reproduction, or distribution of these items is prohibited. To obtain permission to use the scale, including documentation and details on training, scoring and administration, please visit <https://www.imnashealth.com/> or contact Imnas Health directly at <https://www.imnashealth.com/contact> or [info@imnashealth.com](mailto:info@imnashealth.com)

Items are provided in the order of the scales presented in the main paper and not how the WiDI is usually presented.

**PART A**

The following **13 questions** refer to residents' everyday experiences regarding their abilities and capability to do things independently. By "on their own" or "by themself", we mean to do it **independently**, to be left in their room, to be able to decide or make a choice for themself.

When answering these questions, think about the resident in terms of how they have been **[insert time period]**

Has the resident, on their own/by themself…

1. Undertaken personal care during your shift^[[1]](#footnote-1)^? (e.g., using the toilet, bathing/showering, grooming, dressing)
2. Undertaken necessary daily routines during your shift? (e.g., attending mealtimes, eating food, or going to bed)
3. Made decisions in daily activities during your shift? (e.g. choosing what they're going to do; discussing/making decisions around their own health needs/medication)
4. Shown flexibility and movement during your shift? (e.g., walking a short distance, getting up from their bed, maintaining balance, turning over in bed, picking an item up)
5. Had strength/endurance during your shift? (e.g. using multiple spaces in their living environment, navigating (walking, sitting down) around their living area, able to pick up objects from the floor, walk longer distances)
6. Shown co-ordination during your shift? (e.g., manage hand movements, hold objects securely, walk steadily on uneven ground such as grass, gravel, snow)
7. Performed small tasks during your shift? (e.g., pouring water into a glass, stirring tea with spoon, opening/closing of the curtains of their room)
8. Focused on tasks during your shift? (e.g. able to complete tasks, search fully for an item when needed, not be distracted by other tasks)
9. Responded to conditions/situations in the environment during your shift? (e.g., changing clothing appropriate to the temperature, using handrails when needed, moving objects in their room when needed, asking for help)
10. Had personal goals during your shift? (e.g., having something they would like to achieve, committing to activities to achieve personal goals)
11. Been involved in multiple activities during your shift? (e.g., has a variety of things/activities they engage in fully)
12. Managed their own time during your shift? (e.g., able to organise their day, gets prepared for family/friend calls or visits, completes one activity and then moves on to another activity)
13. Been interested in and engages in personal or group activities during your shift? (e.g., involving in any kind of educational activities/training, trying new things or experiences)

**PART B**

The following **8 questions** are about the resident's positive relationships with others and with themself. Positive relationships can be expressed in several ways: **verbally, through body language, facial expressions, or through eye contact.**

When answering these questions, think about the resident in terms of how they have been  **[insert time period]**

To what extent does/is the resident

1. Friendly towards and like their family members, staff or other residents during your shift? (e.g., shows interest in other people, communicates with them positively, starts positive conversations with others, interacts positively with others when in a group, maintains social interest)
2. Had warm and caring relationships during your shift? (e.g., has close and meaningful relationships with family members, staff, other residents)
3. Shown kindness and compassion/sympathy during your shift? (e.g., well-mannered and polite with other residents and staff, recognises and responds to the feelings of others, cares about the needs and situations of others)
4. Engaged in positive communication with others during your shift? (e.g., having appropriate conservations, listening to others, maintaining interest and eye contact during conversations, recognising when to end conversations, not making people feel uncomfortable by getting too close to them).
5. Been able to maintain a positive view of themselves during your shift? (e.g., remaining positive about themselves, feeling overall they have good qualities as a person, accepting that there are positive and negative parts of themselves, accept negative or positive feedback from others)
6. Accepted their own limitations during your shift? (e.g., understanding they face difficulties, able to manage frustrations about their own limitations, show patience/understanding to the difficulties such as disabilities or memory problems)
7. Shown positive belief about their own life during your shift? (e.g., feeling positive about their own abilities, being positive about their life conditions and environment/group they live with, feeling they have control even with difficulties, having the ability to do something difficult and getting pleasure from it)
8. Been kind towards themselves during your shift? (e.g., able to learn positively from mistakes, notice when they do well even with small tasks, showing positive attitudes towards themselves even when they fail to do something)

Items reproduced by Permission of Imnas Health: **© 2025 Imnas Health. All rights reserved.**

1. References to “during your shift” were removed for the community sample. [↑](#footnote-ref-1)
